# Effects of dietary allspice, *Pimenta dioica* powder on physiological responses of *Oreochromis mossambicus* under low pH stress

**DOI:** 10.1186/s40064-015-1520-7

**Published:** 2015-11-24

**Authors:** Sevdan Yılmaz, Ümit Acar, Osman Sabri Kesbiç, Nejdet Gültepe, Sebahattin Ergün

**Affiliations:** Department of Aquaculture, Faculty of Marine Sciences and Technology, Çanakkale Onsekiz Mart University, Çanakkale, 17000 Turkey; Department of Aquaculture, Faculty of Fisheries, Muğla Sıtkı Koçman University, Muğla, 48000 Turkey; Inebolu Vocational School, Sea and Port Management Program, Kastamonu University, İnebolu, 37500 Kastamonu Turkey; Department of Genetics and Bioengineering, Faculty of Engineering and Architecture, Kastamonu University, Kastamonu, 38000 Turkey

**Keywords:** Tilapia, Plant, Supplementation, Biochemistry, Hematology

## Abstract

This study investigated the effects of the supplementation with allspice (0, 5, 10, 15, or 20 g kg^−1^) on the haemato-immunological and biochemical variables in tilapia, *Oreochromis mossambicus* under acidic stress condition. In a 60-day feeding trial, 15 aquariums (80-L) were stocked with 18 fish (20.05 ± 0.10 g) each. Then, acidic stress was achieved by exposing the sampled fish to acidic water (pH 5.5) for 3 days. Allspice supplementation influenced the haematological indices, serum glucose, protein, globulin and innate immune parameters such as respiratory burst activity, lysozyme, and myeloperoxidase activities. In general, at acidic pH decreased circulating red blood cell numbers (RBC), increased mean corpuscular volume (MCV), mean corpuscular hemoglobin (MCH) and the innate immune parameters were observed. On the other hand, the inclusion of allspice prevented an increase in blood glucose MCV and MCH, decreases in albumin, RBC, lysozyme activity and respiratory burst avtivity. In conclusion, this study demonstrated that allspice supplementation at 10 g kg^−1^ for 60 days, has adequate beneficial effects on improvement of haemato-immunological and biochemical status of *O. mossambicus* after stressful management.

## Background

In an outdoor aquaculture system, fish are usually exposed to physical and chemical stressors, such as fluctuations in water oxygen, temperature and pH. Rapid fluctuations in pH are generally more problematic for fish than specific individual pH values (Roberts and Palmeiro 2008). Tilapia is less tolerant to water pH and may develop physiological changes following transfer from neutral water to acidic water (El-Sayed 2006). Low or high water pH may lead to behavioural changes, damage of gill epithelial cells, reduction in the efficiency of nitrogenous excretion and increased mortality of *Oreochromis niloticus* and *O. mossambicus* (Yada and Ito 1997).

Among nutritional antioxidants, it has been reported that vitamins C (ascorbic acid), E (α-tocopherol) and B6 (pyridoxine HCl) play a beneficial role in ameliorating stressful conditions in sea bream (*Sparus aurata*), tilapia (*O. niloticus*) and hybrid catfish (*Clarias macrocephalus* × *C. gariepinus*) (Montero et al. 1999; Pitaksong et al. 2013). In addition, it is known that herb extracts *Rheum palmatum* and *R. rebis* could increase the resistance to high temperature stress in Kutum fish (*Rutilus frisii kutum*) (Babak et al. 2012a, b).

Allspice (*Pimenta dioica*) has been used in traditional folklore medicine for several hundreds of years (Nayak and Abhilash 2008). It can be used to remedy poor appetite, chills, diarrhea, dyspepsia, high blood sugar, and rheumatism (Mars 2007). Allspice has also been shown to have antioxidant (Kikuzaki et al. 1999) and antimicrobial properties (Du et al. 2009). Moreover, some studies indicate significant cytoprotective activities of allspice (Du et al. 2009; Nayak et al. 2008). For this reason, allspice is appearing a good feed additive and it can be used for ameliorating stressful conditions. According to this, the objective of this study was to evaluate the effect of allspice on the haemato-immunological and biochemical status of tilapia, *O. mossambicus* after acidic stress.

## Methods

### Experimental fish and culturing conditions

Healthy cultured *O. mossambicus* (mean weight ± SD = 20.05 ± 0.10 g) were produced in the laboratory of Fisheries Faculty of Çanakkale Onsekiz Mart University (Çanakkale, Turkey). The temperature and dissolved oxygen of the water were measured with a YSI Pro2030 probe, and pH was measured with a HANNA (Model HI 2221; Hanna Instruments, USA) photometer. Total ammonia, nitrite and nitrate were determined by spectrophotometry using Merck test kits and measured by a Optizen POP UV/VIS Spectrophotometer. During the experiment, water quality characteristics (mean ± SE) were as follows: temperature was 27 ± 0.5 °C, pH was 7.2 ± 0.2, dissolved oxygen was 7.5 ± 0.2 mg L^−1^, conductivity was 595 ± 10 uS, total NH_3_ was 0.09 ± 0.02 mg L^−1^, nitrite was 0.03 ± 0.01 mg L^−1^ and nitrate was 1.1 ± 0.3 mg L^−1^ throughout the experiment. Fish experiments were performed in accordance with the guidelines for fish research from the animal ethic committees at Çanakkale Onsekiz Mart University.

### Experimental herb and diets

Allspice (*P. dioica*) seed meal was obtained from Kotanyi, GmbH (Istanbul, Turkey). It was added to the feed at a rate of 0, 5, 10, 15 and 20 g kg^−1^. The feed components of the diets are presented in Table 1. The ingredients were mixed in a mixer. The feed was pressed through a 2-mm die in a pelleting machine, and the pellets were dried in a drying cabinet (40 °C) until moisture dropped to around 10 %. The pellets were crushed into desirable particle sizes and stored at −20 °C until use.

**Table 1:** Formulation of experimental diet (36 % crude protein, 10 % fat)

Ingredients	Concentration (g/kg)
Fish meal^a^	280.0
Soybean meal^a^	320.0
Wheat flour^a^	262.0
Fish oil^a^	65.0
Vitamin–mineral mix^b,c^	40
Starch^d,f^	33.0–13.0
Allspice^e^	0–20

^a^Anchovy fish meal, soybean meal, wheat flour and anchovy fish oil. Sıbal Inc., Sinop, Turkey

^b^Vitamin mix: Vit. A 18,000 IU, Vit D3 2500 IU, Vit. E 250 mg/kg, Vit. K3 12 mg/kg, Vit. B1 25 mg, Vit. B2 50 mg, Vit. B3 270 mg, Vit. B6 20 mg, Vit. B12 0.06 mg, Vit. C 200 mg, folic acid 10 mg, calcium d-pantothenate 50 mg, biotin 1 mg, inositol 120 mg, choline chloride 2000 mg

^c^Mineral mix: Fe 75.3 mg, Cu 12.2 mg, Mn 206 mg, Zn 85 mg, I 3 mg, Se 0.350 mg, Co 1 mg

^d^Wheat starch. Kenton, Ankara, Turkey

^e^Allspice. Kotanyi, GmbH, Istanbul, Turkey

^f^The same diet modified by replacing starch with different amounts of allspice to give 5, 10, 15 and 20 g/kg

### Experimental design and feeding trial

Fifteen 80-L aquarium were stocked with 270 fish (18 fish per aquarium, three replicates). To acclimatize the tilapia to the experimental conditions, the fish were fed the basal diet for 2 weeks. The experimental fish were fed to apparent satiation twice a day for 60 days. Each aquarium was provided with sponge filters connected via airline to a Resun LP-100 air pump. During the experiment, water was exchanged daily at a rate of ~10 % of the total volume.

### Acidic stress

Fish were not fed for 24 h before exposing them to acidic water. All group of fish were subjected to stress. Nine fish from per group (three fish per aquarium) were used to haematological, biochemical and immunological evaluations. An acidic stress was achieved by exposing the sampled fish to acidic water (pH 5.5) for 3 days. Aquarium pH was measured with a digital Hanna instruments pH meter (HI 2221).

### Blood collection

In the experiment, 9 fish on the 60th day were used for blood sampling in control group. To Balance number of fish in groups, 9 fish from each group were excluded from the trial. After the acidic stress experiment, blood sampling was conducted to assess the effects of dietary allspice on haematological variables under stressful conditions. In addition, the blood stress indicators, including blood glucose, serum protein, albumin and globulin, and innate immune parameters of the fish were examined. Fish from each diet anesthetized with clove oil. Blood samples were collected from the caudal vein using a syringe. And the blood was added to tubes containing EDTA 10 % (BD Microtainer^®^, UK). Blood serum was separated by centrifugation (4000×*g*, 10 min) in plastic biochemistry tubes (Kima-vacutest^®^, Italy) and stored at −20 °C until used for biochemical analysis (Bricknell et al. 1999).

### Hematological analysis

Red blood cells (RBC, 10^6^ per mm^3^), hematocrit (Hct, %) and hemoglobin (Hb, gdL^−1^) was determined using the method of Blaxhall and Daisley (Blaxhall and Daisley 1973). RBC was counted with a Thoma hemocytometer using Dacie’s diluting fluid. Hct was determined using a capillary hematocrit tube. Hb concentration was determined by spectrophotometry (540 nm) using the cyanomethahemoglobin method. Mean corpuscular volume (MCV), mean corpuscular hemoglobin (MCH), and mean corpuscular hemoglobin concentration (MCHC) were calculated using the following formulas (Bain et al. 2006).

$$\begin{aligned} & {\text{MCV}}\;(\upmu {\text{m}}^{3} )= [({\text{Hct}},\;\% )\times 10]/({\text{RBC}},\; \times\,10^{6}\,{\text{per mm}}^{3} ), \hfill \\ &{\text{MCH }}({\text{pg}}) = [({\text{Hb,}}\;{\text{g/dL}}) \times 10]/({\text{RBC}},\;\; \times\,10^{6}\,{\text{per mm}}^{3} ), \hfill \\ & {\text{MCHC }}(\% ) = [({\text{Hb,}}\;{\text{g/dL}}) \times 100]/({\text{Hct}},\;\% ).\end{aligned}$$

### Biochemical analyses

Biochemical indices in serum including glucose (GLU), total protein (Tprot), albumin (ALB) and globulin (GLO) were determined using bioanalytic test kits (Bioanalytic Diagnostic Industry, Co) and measured by a shimadzu spectrophotometer (PG Instruments, UK). Serum globulin was determined by the following formula: Globulin = total protein − albumin.

### Immunological analyses

#### Respiratory burst activity (NBT assay)

The respiratory burst activity (RBA) of the neutrophils and monocytes was quantified by the reduction NBT (nitroblue tetrazolium) to formazan as a measure of the production of oxygen radicals. 0.1 ml of the blood from fish of each group was mixed with an equal amount of 0.2 % NBT solution for 30 min at room temperature. After incubation 0.05 ml of solution was taken from the NBT-blood cell suspension and added to a glass tube with 1 ml of N, N diethylmethyl formamide and then centrifuged at 3000×*g* for 5 min. The optical density of the supernatant was measured at 540 nm (Siwicki and Anderson 1993).

#### Lysozyme activity

Serum lysozyme (Lyso) was assessed using the turbidometric assay (Ellis 1990). A suspension of 875 μL of *Micrococcus lysodeikticus* (Sigma, ATCC 4698) at a concentration of 0.2 mg mL^−1^ (in PBS) and was added to 25 μL of serum samples were measured spectrophotometrically at 530 nm after 0.5 and 4.5 min at 25 °C, using a spectrophotometer. A unit of lysozyme activity was defined as the amount of serum causing a reduction in absorbance of 0.001 min^−1^.

#### Myeloperoxidase activity

Total myeloperoxidase (MPO) content in serum was measured according to Quade and Roth (1997) with minor modifications. 30 μL serum was diluted with 370 ml of HBSS without Ca^2^ or Mg^2^ in eppendorf tubes. 100 μL of 0.1 mg/ml 3,3′,5,5′-tetramethylbenzidine dihydrochloride and 0.006 % fresh hydrogen peroxide were added. The reaction was followed kinetically every 50 s for 4.5 min. Reaction velocities were determined as IU, defined as the amount of enzyme required to produce an 0.001 increase in absorbance per minute 0.5 ml of reaction mixture (ΔA 450/min/mL).

#### Statistics

Each value was expressed as mean ± Standart Error (SE) for each of the measured variables. The analyses were performed using SPSS 17.0 (SPSS Inc., Chicago, IL, USA). Statistical significance was established at *P* < 0.05.

## Results and discussion

**Fig. 1:**
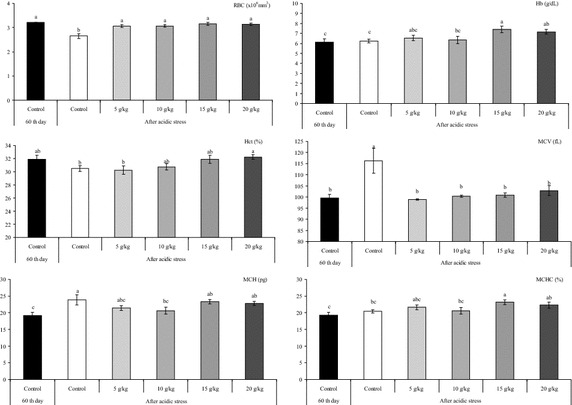
Changes in red blood cells (RBC, ×10^6^ mm^3^), hemoglobin (Hb, g/dL), hematocrit (Hct,  %), mean corpuscular volume (MCV, fL), mean corpuscular hemoglobin (MCH, pg), mean corpuscular hemoglobin concentration (MCHC, %) values under acidic conditions after fed *Oreochromis mossambicus* with allspice supplemented diets for 60 days. Values within the *different superscripts* are significantly different (*P* < 0.05)

Haematological variables are good predictors for explaining the fish health status. The haematological characteristics of healthy fish vary according to internal and external factors (Teixeira et al. 2012; Faggio et al. 2015). The stressful condition significantly decreased RBC in fish that were fed the control diet, except diets that had been supplemented with 5–20 g kg^−1^ allspice (Fig. 1). The results showed that acidic stress condition did not significantly change the levels of Hb, Hct and MCHC (Fig. 1) in control group (*P* < 0.05). However, increasing Hb levels (*P* < 0.05) were obtained in supplemented diets with 15–20 g kg^−1^ allspice (Fig. 1). After acidic stress, Hct percentage was significantly higher (*P* < 0.05) in 20 g/kg allspice group compared with control group (Fig. 1). In addition, the dietary supplementation of 15 g kg^−1^ allspice showed the highest (*P* < 0.05) MCHC value among the all groups (Fig. 1). Fish that were fed with control diet showed significant increase (*P* < 0.05) in MCV and MCH (Fig. 1) under stresfull conditions and supplemented diets with 5–10 and 5–20 g kg^−1^ of allspice had lowering effects, respectively (*P* < 0.05).

In this study, there were signs of an impaired physiological condition caused by acid exposure and significantly changes in RBC, MCV and MCH. Decreased RBC (erythrocytopenia) and increased MCV and MCH were observed in fish after the acidic pH, suggestiong a macrocytic normochromic anemia (Elahee and Bhagwant 2007). This anemia may be due to the inhibition of erythropoiesis and to an increase in the rate of erythrocyte destruction in hemopoietic organs (Adhikari et al. 2004). Our results showed the benefits of supplementation with 10 g kg^−1^ allspice to improve RBC, MCV and MCH after acidic stress. Nayak and Abhilash (2008), also reported that the oral doses (250, 500, 750 mg/kg/day) of allspice protect the haemoglobin from degradation in mice induced by cyclophosphamide. Hematoprotective effect can be related to the high total phenolic content in the leaves or berries of allspice (Nayak and Abhilash 2008; Nayak et al. 2008).

Significant negative associations among unsuitable water quality and fish physiological responses were observed in most of the studies. For example, Nussey et al. (2002), reported that at acidic pH copper and zinc concentrations negatively affected the hematological parameters (RBC, Hb, Hct, and MCV), which can be ascribed to anemic and hypoxic conditions, gill damage and impaired osmoregulation of *O. mossambicus*. Similarly, the significant decrease in the number of RBCs, Hb, Hct and MCV of *Tilapia soarrmanii* after exposure to manganese at pH 5 can be attributed to internal hemorrhaging that was observed (Wepener et al. 1992). Ghanbari and Jami (2012) and Das et al. (2006) also reported reductions in RBC and Hb after exposing carps, *Cyprinus carpio*, *Catla catla*, *Labeo rohita* and *Cirrhinus mrigala* to acidic water pH. Similarly salinity affect the hematological parameters in cultured mullet, *Mugil cephalus* (Fazio et al. 2013).

**Fig. 2:**
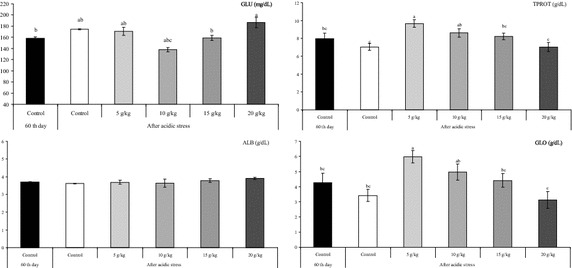
Changes in glucose (GLU, mg/dL), total plasma protein (TPROT, g/dL), albumin (ALB, g/dL) and globulin (g/dL) levels under acidic conditions after fed *Oreochromis mossambicus* with allspice supplemented diets for 60 days. Values within the *different superscripts* are significantly different (*P* < 0.05)

Serum glucose has often been suggested as a useful nonspecific stress indicator (Evans et al. 2003). Our results showed slightly increase (*P* > 0.05) in serum glucose (Fig. 2). However, Pitaksong et al. (2013) reported that the acidic stress (pH 5.5 for 24 h) increased the blood glucose levels in the hybrid catfish (*C. macrocephalus* × *C. gariepinus*) fed control diet. The difference in these results may be due to the different fish species and/or application time used. The increase in serum glucose level and of the acidic pH treatment may be associated with glycogenesis to provide energy for the increased metabolic demands imposed by the acidic water. The change in water pH for fish might have caused the glycogen metabolism in the different organs like the gill and liver (Bhaskar and Govindappa 1985). Bhaskar and Govindappa (1985) reported that at the acidic pH the level of the gill glycogen and free glucose contents were lower in *O. mossambicus*. The glycogen content was elevated, suggesting increased glycogenesis or/and decreased glycogenolysis (Bhaskar and Govindappa 1985), and thus has increase the conversion of liver glycogen to blood glucose to satisfy the greater energy demands of the body under stress (Miron et al. 2008). In this study, supplementation with 10 g kg^−1^ of allspice led to slightly decreasing in blood glucose under stressfull conditions. This can indicate that supplementation of allspice has minimized the stress responses of fish.

Serum total protein, albumin and globulin levels are also thought to be related to a stronger innate immune response in fish (Yılmaz and Ergün 2012). The results showed a slight decrease (*P* > 0.05) in total protein, albumin and globulin (Fig. 2). However, the dietary supplementation with 5 and 10 g/kg allspice increased the level of total protein and globulin in the acidic condition compared with the control group (*P* < 0.05).

**Fig. 3:**
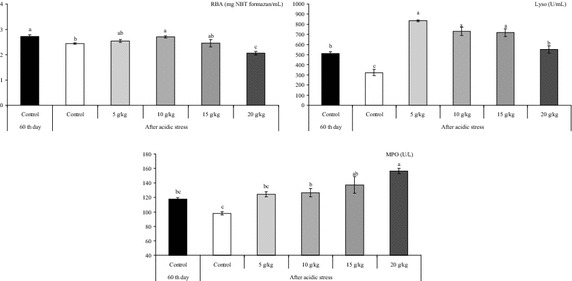
Changes in respiratory burst activity (mg NBT formazan/mL), lysozyme activity (Lyso, U/mL) and myeloperoxidase activitiy (MPO, U/L) values under acidic conditions after fed *Oreochromis mossambicus* with allspice supplemented diets for 60 days. Values within the *different superscripts* are significantly different (*P* < 0.05)

The non-specific defence mechanisms play a key role in maintaining effective disease resistance to a variety of fish pathogens (Kiron 2012). Water environmental stressors, such as temperature, pH, salinity etc. may also be affecting factors pathogenic microorganisms use to subvert the fish’s immune response and cause disease (Dominguez et al. 2005). The stressful condition had the effect of reducing the respiratory burst activity in fish fed control diet (*P* < 0.05). Furthermore, the dietary supplementation with 10 g kg^−1^ allspice significantly increased (*P* < 0.05) the level of respiratory burst activity in acidic condition compared with the control group (Fig. 3). After acidic stress, the lysozyme was decreased in fish fed control diet (*P* < 0.05). The lysozyme level in stressed fish fed 5–20 g kg^−1^ allspice diets were also increased (*P* < 0.05) in acidic stress condition compared with the control diet (Fig. 3). The results showed that acidic stress conditions did not significantly changed (*P* > 0.05) the levels of myeloperoxidase (Fig. 3). However, the stressful condition significantly increased (*P* < 0.05) myeloperoxidase in fish fed 10–20 g kg^−1^ allspice diets compared with those fed control dietcontrol diet (Fig. 3). Similarly, a decrease in respiratory burst activity were reported in white shrimp *L. vannamei* following exposure to pH 6.5 after 6–72 h indicating a decrease in the respiratory burst that might be related to a decrease in the production of H_2_O_2_ and leading to decreased catalase and peroxidase activities (Li and Chen 2008). Like the present study, Pitaksong et al. (2013), reported that the acidic stress (pH 5.5 for 24 h) had the effect of reducing the lysozyme activity in hybrid catfish (*C. macrocephalus* × *C. gariepinus*) fed diets deficient of vitamin C and E for a period of 8 week. In contrast to our results, the increase in lysozyme activity by low pH (4.0) was reported in *O. niloticus* for a long term (2 weeks) acid induced (Dominguez et al. 2005). The difference in these results may be due to the different values of the pH used. Additionally, it is possible to suggest an interaction effects among time and pH on immune function.

## Conclusion

The results of the present study demonstrated that allspice supplementation at 10 g kg^−1^ for 60 days, has adequate beneficial effects by improving the haemato-immunological and biochemical status of *O. mossambicus* after acidic stress.

## Abbreviations

RBC: red blood cell
MCV: mean corpuscular volume
MCH: mean corpuscular hemoglobin
EDTA: ethylenediaminetetraacetic acid
Hct: hematocrit
Hb: hemoglobin
MCHC: mean corpuscular hemoglobin concentration
GLU: glucose
Tprot: total protein
ALB: albumin
GLO: globulin
RBA: Respiratory burst activity
NBT: nitroblue tetrazolium
Lyso: serum lysozyme
MPO: myeloperoxidase
HBSS: Hanks balanced salt solution
